# Survey and evaluation of mutations in the human KLF1 transcription unit

**DOI:** 10.1038/s41598-018-24962-3

**Published:** 2018-04-26

**Authors:** Merlin Nithya Gnanapragasam, John D. Crispino, Abdullah M. Ali, Rona Weinberg, Ronald Hoffman, Azra Raza, James J. Bieker

**Affiliations:** 1 Department of Cell, Developmental, and Regenerative Biology, Mount Sinai School of Medicine, New York, NY 10029 USA; 2 Department of Medicine, Northwestern University, Chicago, IL 60611 USA; 3 Department of Medicine, Columbia University Medical Center, New York, NY 10032 USA; 4 Cellular Therapy Laboratory, New York Blood Center, New York, NY 10065 USA; 5 Department of Medicine, Mount Sinai School of Medicine, New York, NY 10029 USA; 6 Tisch Cancer Institute, Mount Sinai School of Medicine, New York, NY 10029 USA; 7 Black Familly Stem Cell Institute, Mount Sinai School of Medicine, New York, NY 10029 USA; 8 Mindich Child Health and Development Institute, Mount Sinai School of Medicine, New York, NY 10029 USA

## Abstract

Erythroid Krüppel-like Factor (EKLF/KLF1) is an erythroid-enriched transcription factor that plays a global role in all aspects of erythropoiesis, including cell cycle control and differentiation. We queried whether its mutation might play a role in red cell malignancies by genomic sequencing of the KLF1 transcription unit in cell lines, erythroid neoplasms, dysplastic disorders, and leukemia. In addition, we queried published databases from a number of varied sources. In all cases we only found changes in commonly notated SNPs. Our results suggest that if there are mutations in KLF1 associated with erythroid malignancies, they are exceedingly rare.

## Introduction

Erythroid Krüppel-like Factor (EKLF/KLF1) is a red cell-enriched, zinc finger DNA binding protein that interacts with its cognate 5′CCMCRCCCN3′ element at target promoters and enhancers^1^. Its roles in ß-like globin gene regulation during terminal erythroid differentiation have been well-established using genetic, biochemical, and molecular approaches^2,3^. Specific functional properties and expression characteristics of EKLF, along with recognition of its surprisingly broad role prior to and during red cell differentiation (reviewed in^4–8^), provide the conceptual basis for the present study.

First, single amino acids and their modifications are critically important for EKLF protein-protein interactions and its function as an activator or repressor^9–12^, raising the possibility that mutations at these sites or within their consensus sequences could have dramatic functional effects on gene expression control.

Second, subtle altering of EKLF cellular levels can change the erythroid cell cycle status from proliferation to differentiation, an effect mediated, at least in part, by its direct activation of the p21, p18, p27, and E2f2 genes^13–17^. Induction of p21 is reminiscent of a similar up-regulation that has been observed with KLF4^18^ and with KLF6^19^, known tumor suppressors that function in a p53-independent manner and that are frequently inactivated or downregulated in human cancer^20^.

Third, EKLF mRNA is highly restricted in its expression pattern during development to erythropoietic organs such as the yolk sac, fetal liver, adult bone marrow, and red pulp of the spleen^1,21^. Although most abundant in the erythroid cell, EKLF is also highly expressed in the megakaryocyte/erythroid progenitor (MEP)^22^. Its level is downregulated as MEPs differentiate towards the megakaryocytic lineage yet remains high in the erythroid lineage^22^. By using both gain- and loss-of-function approaches, we^12,22^ and others^23–25^ have found that the expression levels of EKLF impacts the bipotential lineage decisions that are made by the MEP; specifically, EKLF inhibits the formation of megakaryocyte colony and cell numbers while at the same time stimulating erythroid differentiation.

Fourth, there are now links between KLF1 mutation and altered mammalian hematology^26–28^. For example, the semi-dominant mouse mutation Nan (neonatal anemia), which presents with hereditary spherocytosis, was mapped to a single amino acid change (E339D) within the second zinc finger of EKLF^29–31^. The mutation alters the DNA binding specificity of EKLF such that it no longer binds promoters of a subset of its DNA targets^29^. In addition, recognition of a novel target DNA sequence by Nan-EKLF leads to ectopic expression of genes not normally expressed in the red cell, yielding a neomorphic phenotype with cellular and systemic consequences^32,33^.

Excitingly, this murine mutation has converged with human disease, particularly a subtype of congenital dyserythropoietic anemia (CDA)^34–39^, where the same amino acid is altered (albeit to another charged residue, lysine) in human EKLF/KLF1. These patients are severely anemic with highly elevated HbF and reticulocyte levels, membrane abnormalities, severe hemolytic anemia, erythroid hyperplasia with dyserythropoiesis, splenomegaly, and growth delay^36^. KLF1/E325K is recognized as a characteristic feature of CDA type IV^40^.

Additionally, the regulation of some genes are uniquely sensitive to haploinsufficient levels of KLF1 (Lu, Bcl11a, HbA2), leading to altered genetic expression patterns and hematologic parameters in humans, of which hereditary persistence of fetal hemoglobin (HPFH) is particularly relevant to clinical outcome^4,5,26,41–43^.

Collectively these functional properties suggest that genetic mutation or altered levels of KLF1 may also be a causative factor for a specific subset of hematopoietic disease. We focused our attention on two types of disorders. Myeloproliferative neoplasms (MPN) are chronic hematological malignancies that yield an excessive proliferation of blood cells with normal differentiation^44,45^. On the other hand, unrestricted proliferation and impaired differentiation are characteristic of acute myeloid leukemia (AML)^46,47^. Our test hypothesis is that dysregulation of KLF1 function may contribute or lead to either of these human malignancies.

## Results

### Chromosomal associations

19p13.13, the chromosomal locus of KLF1, has been associated with variation in blood cell traits in meta-analysis studies^48^. Recent studies provide an extensive catalogue of SNP variants of consequence for red cell parameters^49^. Perusal of the data enable us to extract the ones most relevant to KLF1 as summarized in Table 1, suggesting these KLF1 gene variants are significantly associated with altered MCH, MCV, and MCHC red cell indices, and RBC and RET numbers. In combination with studies summarized in the Introduction, we felt justified to address whether mutagenic changes in KLF1 might also be associated with aberrant or malignant red cell parameters.

**Table 1:** KLF1 loci associated with RBC traits.

Associated Blood Index	rsID	BP (GRCh37)	REF/ALT	MAF (%)	Univariable Analysis			
					Estimate of Additive Allelic Effect	Standard Error of Estimator	−log_10_ P	Unadjusted R2
RET#	rs3817621	12998205	G/C	23.9	−0.038	0.0042	18.6	0.000527
RET%	rs3817621	12998205	G/C	23.9	−0.046	0.0042	27	0.000776
RBC#	rs56397034	13000550	G/C	38.9	−0.049	0.0036	41.3	0.001166
MCV	rs56397034	13000550	G/C	38.9	0.068	0.0036	78.5	0.002203
MCH	rs56397034	13000550	G/C	38.9	0.071	0.0036	85.3	0.002411
MCHC	rs11085824	13001547	A/G	37.6	0.029	0.0035	15.8	0.000407

SNPs (rsID) at or near the KLF1 gene associated with red blood cell indices are tabulated with respect to chromosome 19 location (Ch37), percent minor allele frequency (MAF), and nucleotide change compared to the reference genome (REF/ALT). Univariable analysis indicates the direction and significance of the allelic effect on the index parameter. Data from^49^.

### Genomic analyses of KLF1 in selected populations

The complete human KLF1 transcription unit is only 3.5 kB^50^, enabling us to interrogate its proximal promoter, 5′ UTR, introns, exons, and 3′ UTR by eight overlapping amplimers. The proximal promoter contains highly conserved transcription factor binding sequences that are critical for its expression in erythroid cells^51–55^. To begin our evaluation of human KLF1 genomic status, we focused first on sequencing human leukemia cell lines that retain erythroid and/or megakaryocytic features^56,57^. These lines are derived from CML, AML-M6 or -M7 patients (F36P, HEL, JK1, K562, KMOE2, KU812, LAMA84, OCIM1, TF1), and include those with mixed erythroid/megakaryocytic features (CMK, KG1, Meg01). We conclude from genomic sequence comparison of these lines to the 1000 Genomes project^58,59^ that the KLF1 genomic changes observed represent known single nucleotide polymorphisms (SNPs), but no novel mutations (Table 2).

**Table 2:** SUMMARY of genomic sequence analyses.

Sample Type	rsID	BP (GRCh38)	number	REF/ALT	MAF (%)	Location	Predicted effect***
cell lines	rs3817621	12887391	8	G/C	32.5	**promoter (−188)	
	rs112631212	12886115	1	T/G	1.44	**M39L-class 1	likely benign
	rs2072597	12885926	10	A/G	44.4	**S102P-class 1	likely benign
	rs16978757	12884608	1	G/A	5.61	3′UTR	
	rs16978754	12884589	1	T/C	5.59	3′UTR	
MPN	rs115672848	12888141	1	C/T	*0.14	promoter (−938)	
	rs3817621	12887391	5	G/C	32.5	**promoter (−188)	
	rs79334031	12887288	2	C/T	1.58	**promoter (−85)	
	rs112631212	12886115	7	T/G	1.44	**M39L-class 1	likely benign
	rs2072597	12885926	12	A/G	44.4	**S102P-class 1	likely benign
	rs2072596	12885686	3	A/G	4.95	**F182L-class 1	likely benign****
	rs16978757	12884608	1	G/A	5.61	3′UTR	
	rs16978754	12884589	1	T/C	5.59	3′UTR	
MDS	rs201870270	12887780	1	delA	*0.8	promoter (−577)	
	rs3817621	12887391	15	G/C	32.5	**promoter (−188)	
	rs79334031	12887288	4	C/T	1.58	**promoter (−85)	
	rs112631212	12886115	1	T/G	1.44	**M39L-class 1	likely benign
	rs2072597	12885926	21	A/G	44.4	**S102P-class 1	likely benign
	rs182276666	12885919	1	G/A	*0.08	**A104V-class 1	likely benign
	rs2072596	12885686	2	A/G	4.95	**F182L-class 1	likely benign****
	rs16978754	12884589	2	T/C	5.59	3′UTR	
AMKL	rs3817621	12887391	1	G/C	32.5	**promoter	
	rs2072597	12885926	5	A/G	44.4	**S102P-class 1	likely benign

Tabulation of all KLF1 SNPs (rsID) found in the present study, grouped together based on cell types as described in the Results. Included are the number of examples of each change, along with chromosome 19 location (Ch38), nucleotide change compared to the reference genome (REF/ALT), and percent minor allele frequency (MAF). Location with respect to the KLF1 transcription unit (promoter, coding region, 3′UTR) are as indicated, along with the amino acid change. “Class 1” refers to the tabulation in^26^, indicating that any amino acid change is likely benign, a conclusion supported by the “Predicted effect” based on other criteria^66,67^. KLF1 transcription initiation is at BP = 12887203 in Ch38 (based on^50,54^).

*rare (<1%); **noted previously in reference^26^ as implicated in hypomorphic KLF1 expression; ***based on references^66,67^; ****PolyPhen suggests ‘possibly damaging’ due to cross-KLF family conservation of F (phenylalanine) at this position, possibly by decreasing its stability^98^.

We then directly assessed primary human DNA samples from a selected cohort of patients. Given that KLF1 levels may be playing a directive role in erythroid/megakaryocyte bipotential decisions, we focused on myeloproliferative neoplasms (MPNs) as cells whose aberrant properties might result from expression of mutated KLF1. MPNs are a heterogeneous group of clonal hematological malignancies that are characterized by hypercellular bone marrow, panmyelosis, and a gradual evolution to myelofibrosis (MF) and acute leukemia^60,61^. These disorders are clonal and yield an excessive proliferation of blood cells that exhibit normal differentiation^44^. We were particularly interested in two subtypes of MPNs: polycythemia vera (PV) and essential thrombocythemia (ET), which represent abnormalities in the proliferation of erythroid and megakaryocytic lineage respectively^45^. We therefore sequenced genomic DNA from a collection of individuals with PV (eighteen), ET (eleven), and MF (five) samples. No novel mutations were identified, only SNPs (Table 2). Although rs115672848 is a rare variant, we found no evidence for its selective enrichment.

Many KLF1 target genes overlap with the expression signature identified in the differential analysis of myelodysplastic syndrome (MDS) patients that vary in their response to lenalidomide treatment^62^. We hypothesized that the mutational status of KLF1 may provide a mechanistic basis to explain the differential expression signature in these patients, and potentially predict whether they will respond to lenalidomide. We analyzed 26 samples each of responders and non-responders. All variants identified in these samples were known SNPs (Table 2), and none partitioned significantly to either of the two differentially responding groups. There were no significant difference in clinical parameters between the most common rs3817621 (p = 0.44) and rs2072597 (p = 0.53) SNPs. In addition, while rs201870270 and rs182276666 are rare variants, they are not selectively enriched.

We next examined ten acute megakaryoblastic leukemia (AMKL) samples^63^, including four from patients with Down syndrome (DS) and six from those without DS. AMKL is an aggressive form of leukemia where a majority of the expanding cells are abnormal megakaryoblasts^64^, and whose DNA methylation patterns are distinct between DS and non-DS cells^65^. Again, all samples contain known KLF1 SNPs (Table 2).

**Figure 1:**
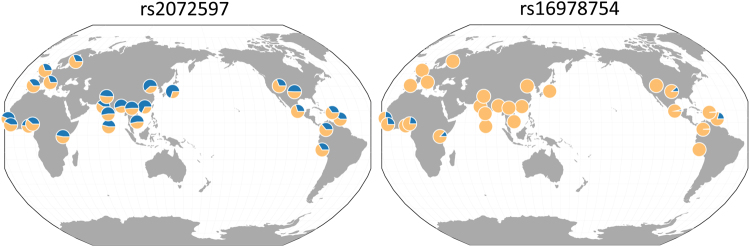
Geographical distribution^68,69^ of two KLF1 SNPs from Table 2 as examples of widely dispersed (rs2072597; >40%) or limited (rs16978754; ~5%) MAFs. Blue pie conveys a given MAF percentage out of 100% across the indicated global populations. rs16978754 is common (~20%) only in the Gambian, Sierra Leone, and Nigerian populations on the African continent, and beyond is commonly detected only in the African Caribbean and African American populations; otherwise it is not detectable.

As indicated in Table 2, many of these variants have been noted before^26^. The ones that result in non-synonymous coding changes are predicted not to affect KLF1 function^66,67^ (with the possible exception of rs2072596 as highlighted in Table 2). However, it is noteworthy that a geographical analysis^68,69^ shows that, unlike ones whose variation is common and universal (e.g., rs2072597), some with an overall low frequency (~5%) are nonetheless highly enriched (~20%) prevalently and exclusively in selected genetic or geographic sub-populations while completely absent in all others (e.g., rs16978754) (Fig. 1).

### Alternative analyses

Given our hypothesis, we were surprised by the absence of mutations associated with our target samples/sources. To expand our analysis, we also considered an *in silico* approach and queried published data from whole genome and exome analyses. These global analyses enable tabulation of altered genes to be accumulated in an unbiased manner. Consistent with our own directed sequencing studies, perusal of recent MPN^70,71^, acute erythroleukemia (250 samples total in two studies^72,73^), and AML tabulations^74^, along with those derived from The Cancer Genome Atlas (TCGA) data sets^75,76^ do not reveal a role for KLF1 in any case in these blood cancers. KLF1 is not one of 142 driver genes identified from an analysis of 1699 pediatric leukemias and tumors^77^. An shRNA screen of AML cells lines also did not implicate a role for KLF1^78^, and KLF1 does not appear in differential analyses related to predicting therapy resistance in AML^79^. Examination of COSMIC data (v83)^80^ indicates that, out of 3478 curated hematopoietic and lymphoid samples, only a single coding sequence variant was observed (F27V) in a CLL patient^81^, one predicted to be benign^66,67^.

**Figure 2:**
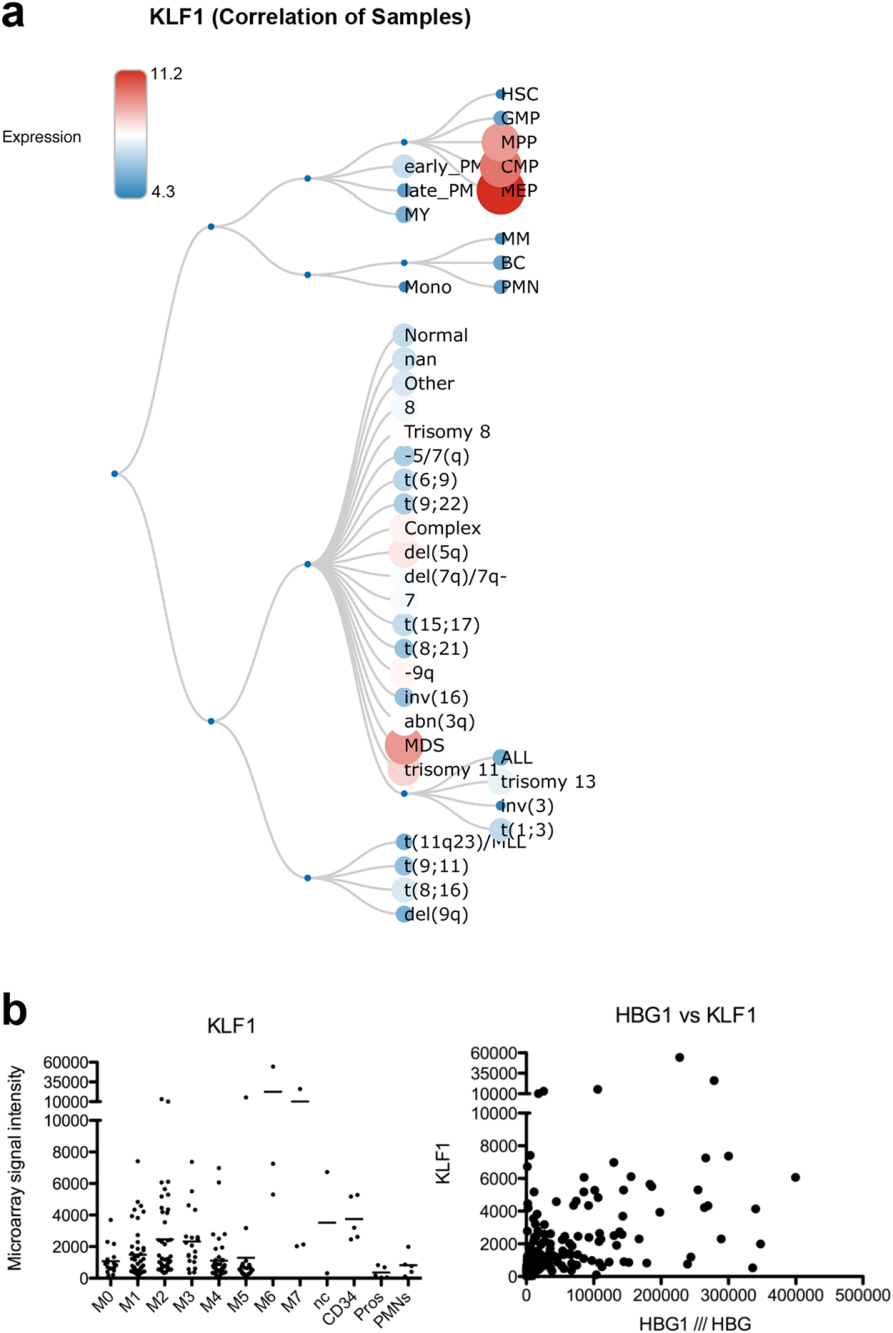
Variation of KLF1 levels across aberrant erythroid sources. (**a**) BloodSpot (^82^; http/servers.binf.ku.dk/bloodspot/?gene=KLF1&dataset=MERGED_AML) analysis of relative KLF1 levels in samples across dysplastic and leukemic sources, as well as that seen during normal hematopoiesis (concordant with murine studies^22^). (**b**) *Left* - Analysis of KLF1 expression in AML samples derived from a range of subtypes (M0-M7), also compared to CD34, promyelocytes (pros) and polymorphonuclear leukocytes (PMNs). nc = not categorized. *Right* – Graph showing lack of correlation between KLF1 and HBG levels in the same set of AML samples. Note that HBG expression within the five highest-expressing KLF1 samples vary tremendously.

We finally considered whether variation in levels of KLF1, rather than a mutated form, might be correlated with a particular blood cancer. This hypothesis arises from two bits of data. One, it is known that some genes are uniquely sensitive to haploinsufficient levels KLF1 (reviewed in^4,26^). Two, KLF1 levels vary considerably across various malignancies as judged by tabulation of RNA sequence datasets^82^ (Fig. 2a). To further address this idea we queried a series of 200 AML samples to see whether KLF1 levels correlate with a particular AML subtype^83^. We were surprised by two observations (Fig. 2b). First, there is a wide range in KLF1 expression within the AML M6 and M7 categories that generally would be anticipated to express elevated levels of KLF1. Second, other AML subtypes, including M0-M5, contain samples that express similar high levels of KLF1. This was not expected as KLF1 is most highly enriched in erythroid, not myeloid, cells. Clearly, normal cellular and genetic control mechanisms are altered in many of these patient samples, as KLF1 levels do not even correlate with HBG expression (Fig. 2b), a target that is normally repressed by KLF1 and whose levels are indirectly proportional to that of KLF1^42^. We conclude there is no apparent correlation between KLF1 levels and malignancy status across a range of AML malignancy subtypes.

## Discussion

Given the molecular and biochemical properties of KLF1, along with its regulation of selected downstream target gene expression, it remains surprising that our study did not uncover any mutations associated with erythroid dyplasia or malignancy. A simple explanation is that an insufficient number of samples have been analyzed. Although this possibility cannot be excluded, we directly analyzed nearly 150 samples, and in addition perused numerous databases without success. In any case, other explanations come to mind.

For instance, we have not considered whether the epigenetic status of the KLF1 gene may be playing a role in its regulation that may be of consequence to malignancy. For example, 5mC modification at the KLF1 locus inversely correlates with its expression when compared across a number of cell types^84,85^. In addition, the level and extent of 5hmC modification at the KLF1 locus is also inversely correlated with its expression as the CD34+ cell differentiates to the mature erythroid cell^86^. Intriguingly, the KLF1 gene exhibits a synergistic “type III/cluster 3” pattern of expression control such that KLF1 transcript levels are dramatically increased in Tet2/Dnmt3a double knockout cells^87^. Of relevance to the present discussion, increased KLF1 levels in a subset of AML is only seen in samples from patients with mutations in both genes^87^. The DNA modification locales overlap regions demonstrated to be important for KLF1 expression control^7,54,55^. These studies demonstrate that the DNA modification status of controlling regions in the KLF1 gene is important for establishing its optimal level of expression. However, the causative versus correlative nature of KLF1 gene epigenetic modification and aberrant erythropoiesis will remain challenging to tease out.

One final explanation for our findings is that KLF1 functions during late stages of erythropoiesis, which may circumscribe any search for an effect at early stages^8^. In other words, it is known that terminal maturation of erythroid cells, particularly at the transition from orthochromatic to reticulocyte stage, is completely dependent on KLF1^17^. Part of the explanation for this requirement is KLF1 regulation of cell cycle inhibitors such as p18 and p27 specifically at this late stage. It is notable that these genes are not dependent on KLF1 at an earlier stage, for example in proliferating erythroblasts; indeed, there are no cell cycle differences when comparing such cells from WT vs KLF1-null^17^. Given that the blood cell disorders that we tested exhibit unrestricted proliferation, it remains possible that mutated KLF1 would not have a causative effect on cell cycle in this context in any case.

In spite of these considerations, we are still left with the example of the monoallelic mutation in KLF1 that leads to CDA type IV, with its dominant effect on erythroid cell properties, including proliferation^34,36,39^. Nonetheless, our results suggest that if there are any KLF1 mutants implicated in erythroid malignancy, they are quite rare.

## Methods

K562 cells were from our original lab stock^88^; all other cell lines were purchased from either the ATCC or DSMZ. K562 cells merit additional discussion. There are inconsistencies in the literature as to whether KLF1 is expressed in this cell line, with some studies indicating low to nil^50,88,89^, and others showing detectable levels^90^. There is a large body of work on its use as a cotransfection reporter line whose utility is dependent on lack of KLF1 expression (studies that began with^88^). This line was established decades ago^91^, and early on was noted to exhibit variability^92,93^. We have noted major differences in transfection efficiency upon comparing our lab K562 stock with the ATCC K562 line (unpublished observations) although we did not find any KLF1 genomic sequence differences. We suggest labs are working with dissimilar isolates, differing in levels of GATA1 and/or KLF1^94^.

Patient samples were procured after informed consent and IRB approval within the individual institutions (Mount Sinai School of Medicine, Columbia University Medical Center, New York Blood Center, Northwestern University School of Medicine). All methods were performed in accordance with the relevant institutional guidelines and regulations. Mononuclear cells from MPN patients were provided by the Myeloproliferative Disorders Research Consortium Tissue Bank Core C.

Genomic DNA was isolated from all samples using a Qiagen DNeasy Blood & Tissue Kit. PCR primers spanning the complete KLF1 transcription unit were used to amplify eight overlapping regions across the locus (Supplemental Table 1). These were individually sequenced in both directions (with their corresponding PCR forward and reverse primer) using a 96-well format (Macrogen USA). With regards to comparison of clinical parameters in MDS samples, for continuous variables satisfying the normality assumption, a two-tailed unpaired t test was used.

Discovery of any nucleotide change(s) followed alignment to the GRCh38 reference sequence using Vector NTI ContigExpress software (ThermoFisher Scientific). SNPs and any associated parameters were identified from the 1000 Genomes databases^58,59,68^. Other datasets queried included BloodSpot^82^, cBioPortal^95^, GGV^69^, UK10K^96,97^, COSMIC^80^, and NCBI public resources.

## Electronic supplementary material

Supplementary Table 1
