# Supplementary material for: Survey and evaluation of mutations in the human KLF1 transcription unit
Source: Sci Rep. 2018 Apr 26;8:6587. doi: 10.1038/s41598-018-24962-3 (PMC5920080; doi:10.1038/s41598-018-24962-3)
Supplement: Supplementary file 1 — Supplementary Table 1 [file 41598_2018_24962_MOESM1_ESM.pdf]

Supplementary Information

Survey and evaluation of mutations in the human KLF1 transcription unit

Merlin Nithya Gnanapragasam[1], John D Crispino[2], Abdullah M Ali[3], Rona Weinberg[4], Ronald Hoffman[5], Azra Raza[3], and James J Bieker[1,6,7,8]

[1]Department of Cell, Developmental, and Regenerative Biology, Mount Sinai School of Medicine, New York, NY 10029. [2]Department of Medicine, Northwestern University, Chicago, IL 60611. [3]Department of Medicine, Columbia University Medical Center, New York, NY 10032. [4]Cellular Therapy Laboratory, New York Blood Center, New York, NY 10065. [5]Department of Medicine, Mount Sinai School of Medicine, New York, NY 10029. [6]Tisch Cancer Institute, [7]Black Familly Stem Cell Institute, [8]Mindich Child Health and Development Institute, Mount Sinai School of Medicine, New York, NY 10029

Supplemental Table 1
KLF1 genomic PCR primer pairs

Name	forward	reverse
hProm3	CTTGATCCAACGGTCCTATCC	AGCCAAGTCAAATATCAAGGGT
5’UTR/Exon1	GCCCCCGCCGCCTTCTTTGGAGACCCAATGTC	CCGAAATAGATCACACTTAGAACC
Int1A	ACACAGGATGACTTCCTCAAGGT	CCAGAACATCCCTCTCCTTCC
Int1B	GCCCCCGCCGGGCTCACAGACAATCTTCCAGA	CATGTCCTGCGCCTCTTC
Ex2A	AAGCCTCTGCGTCAGAGTGTCC	GGAAGTAGCCACCCGAGGAG
Ex2B	TCGGAGGATCACTCGGGTTGG	GGGCTGGGACTAGGATGAACAAAGTG
Int2/3'UTR	CCACCTGAAGGCGCATCTG	ATCTCTGTGTGGCTCCCTGTG
3'UTR	CATGAAGCGCCACCTTTGAG	GGGTGTCACTGTAGTTTAGG
